# Epidemiological characteristics and forecasting incidence for patients with breast cancer in Shantou, Southern China: 2006–2017

**DOI:** 10.1002/cam4.3843

**Published:** 2021-03-16

**Authors:** Huang Lin, Lei Shi, Jiachi Zhang, Jinchan Zhang, Chichen Zhang

**Affiliations:** ^1^ Department of Prevention and Health Care Shantou Central Hospital/Affiliated Shantou Hospital of Sun Yat‐sen University Shantou China; ^2^ Department of Health Management, School of Health Services Management Southern Medical University Guangzhou China; ^3^ Department of Medical Dispute Maternal and Child Health Hospital Heyuan China; ^4^ Department of Health Management Nanfang Hospital, Southern Medical University Guangzhou China; ^5^ Institute of Health Management, Southern Medical University Guangzhou Guangzhou China

**Keywords:** Breast cancer, China, epidemiological characteristics, forecasting, incidence

## Abstract

This study aimed to explore the epidemiological characteristics of breast cancer and establish an Exponential Smoothing (ETS) and Autoregressive Integrated Moving Average (ARIMA) models to predict the development of incidence in Shantou. This study has a large sample size, strong representativeness, and wide‐ranging and comprehensive medical insurance information, which can fill the gaps in basic epidemiological research on breast cancer in Shantou. Successful completion of this study is a helpful tool to understand the epidemiology of Guangdong Province and Southern China. This study also provides data and scientific references for the government and future research on breast cancer prevention and control. This retrospective study was conducted to describe the epidemic intensity, epidemic distribution, and epidemic trend of breast cancer in Shantou, Guangdong Province, from 2006 to 2017, gathered from the Shantou's Medical Security Bureau covers the whole districts of Shantou. ETS and ARIMA models were used to describe the regional distribution, time distribution, and population distribution of breast cancer in Shantou. Moreover, based on the ARIMA model and ETS model, the incidence trend of breast cancer was predicted during 2018–2022. This study included 5,681 breast cancer patients, majority of whom were aged 50–59 years. The male‐to‐female ratio of the breast cancer patients was about 1:107 (the same ratio of the insured population was 1:1). Female patients accounted for 98.61% of the total insured population. The incidence and mortality rates of female breast cancer were 16.42/100,000 and 0.66/100,000, respectively. Based on the ARIMA model or ARIMA and ETS model, a gradually decreasing trend in the incidence of breast cancer is expected in the future. Comparing the performances of the ARIMA model and ETS model, ARIMA (4, 0, 1) (0, 1, 0) model had a lower the root mean squared error and the mean absolute percentage error than ETS (M, N) model. This population‐based retrospective study showed that the high‐risk age for the age‐specific incidence of female breast cancer was 50–55 years. It is recommended that healthcare administration should strengthen program awareness and education regarding breast cancer prevention and control. It is also possible that feasibility of extrapolating the current methodology to other future studies or broader populations in which the cancer registry data are not available.

## Graphical abstract

This is the first population‐based retrospective study of breast cancer in Shantou, Guangdong Province of Southern China in the past 10 years. The result of the age‐specific incidence of female breast cancer indicated that the high‐risk age was from 50 to 55 years. It is recommended that the health administration strengthen the publicity and education regarding the prevention and control of breast cancer.

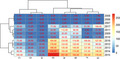

## 1 INTRODUCTION

### 1.1 Epidemiological characteristics of breast cancer

Breast cancer is the most common malignant cancer among women worldwide, particularly in less developed countries.^1^, ^2^, ^3^ Moreover, substantial studies have reported the epidemiological characteristics of breast cancer. In 2018, global cancer statistics showed a total of 18.1 million new cancer cases and 9.6 million cancer deaths.^1^ DeSantis *et al* indicated that the breast cancer incidence rate was rising by 0.3% per year.^4^ International Cancer Research Agency has predicted the rapid increase of cancer cases in the world, with the highest cancer incidence in developing countries in Africa, Asia, and Central and South America.^5^ The incidence rates were 24.5/100,000, 29.3/100,000, and 22.4/100,000 in Africa, North Africa, and Sub‐Saharan Africa, respectively.^6^ Among Asian countries, Israel has the highest incidence and Pakistan has the highest mortality rate associated with breast cancer.^3^

In China, breast cancer morbidity and mortality rate has seen a rapid increase in the past two decades with significant regional characteristics: the incidence was significantly higher in the eastern coastal urban region.^7^, ^8^, ^9^ A Chinese study indicated that breast cancer is associated with the highest incidence and death rate among women at 24.20% and 15.00%, respectively.^5^ According to the latest data of China National Cancer Center, there were 3.929 million new cancer cases and 2.338 million cancer deaths in 2015.^10^ The latest data showed that about 3.804 million new cancer cases (incidence rate 278.07/100,000) and 2.296 million cancer deaths (mortality rate 167.89/100,000) occurred in China, and breast cancer had the highest morbidity rate among Chinese females. Moreover, breast cancer incidence was 40.99/100,000 and mortality was 6.95/100,000 in Guangdong Province, which is the highest among all provinces.^11^ Shantou City, Guangdong Province, is located in the southeast coastal margin of China, which is an area with a high incidence of breast cancer with unique human factors. Since the reform and initiation, Shantou City was one of the special economic zones that experienced rapid social and economic development, improvement of local residents’ income level, as well as significant changes in residents’ dietary habits and lifestyle. In addition to the aggravations associated with an aging population, malignant tumors have become the major diseases affecting the health of local residents. However, in the past 10 years, there have been no epidemiological studies on breast cancer in Shantou.

### 1.2 Prevention and control strategy of breast cancer

Many studies have explored the prevalence, prevention, treatment, prognosis, and intervention measures for breast cancer and obtained remarkable results. However, the exact cause of breast cancer has not yet been fully understood, and the epidemiological analysis and primary prevention still need to be improved. Interventions involving a change in lifestyle or behavior (weight reduction, physical activity, and limited alcohol consumption) have reduced the overall risk of breast malignancy.^12^, ^13^, ^14^

Chen *et al*. proposed that with the growing burden of cancer in China, it is necessary to actively promote cancer registration, as well as expand its scope and formulate prevention and control measures.^15^, ^16^ The scholars also emphasized that measures to ensure early detection, diagnosis, and treatment of cancer should be implemented to reduce the incidence and mortality rate of cancer and improve the survival and quality of life of the patients.^15^ Zeng *et al*. showed that four ways to effectively reduce the mortality rate of cancer are as follows: (1) Prevention, (2) Combination of prevention and treatment, (3) Focusing on the three “early” measures (early detection, early diagnosis, and early treatment), (4) Overcoming three hurdles (etiological prevention, early diagnosis and early treatment, comprehensive prevention, and treatment).^17^

In recent years, free cancer screening programs (cervical cancer and breast cancer) have been carried out in China.^17^ Although, the efficacy of early diagnosis and treatment of breast cancer has already been certified, the influence of population characteristics, geographical conditions, lifestyle, and economic factors limited the promotion and popularization of cancer screening programs.^8^ The principle of “prevention first” should be adhered to and precautionary measures should be emphasized.^18^

### 1.3 Added value of this study

The Chinese National Cancer Registration Center was established in 2002. There are 574 cancer registries in China, covering 438 million people and accounting for 31.5% of the national population in 2018. With the promotion of cancer registration and follow‐up projects, Guangdong Province has gradually expanded the coverage of cancer registration areas since 2009. At present, 18 cancer registration centers have been established, covering the population of 26 million households and accounting for 29.52% of the province's population. However, as of the end of 2020, the whole population of the Shantou area has not been covered, as the difference between cancer registration and the actual local cancer burden has been unknown for a long time. At present, there is no special report on the prevalence of breast cancer in Shantou City.

To our knowledge, this study is the first population‐based retrospective analysis of breast cancer conducted in Shantou, Guangdong Province of Southern China in the past 10 years. This study has a large sample size, strong representativeness, and wide‐ranging and comprehensive medical insurance information, which can fill the gaps in basic epidemiological research on breast cancer in Shantou. Successful completion of this study is a helpful tool to understand the epidemiology of Guangdong Province and Southern China.

### 1.4 Aims and prospects

(1) To fill the gap in knowledge regarding breast cancer epidemiology in Shantou.

(2) To explore the current status of the breast cancer epidemic, establish a trend prediction model, and provide effective prevention and control measures to reduce the intensity of the breast cancer epidemic in Shantou, Southern China.

(3) To provide useful data regarding the breast cancer epidemic in Shantou, Southern China for analysis and comparison with those of other populations in China, Asia, and Western countries.

(4) To provide a scientific reference for future research on breast cancer investigation and prevention in Shantou, other areas in China, and other countries.

## 2 METHODS

### 2.1 Design

This study used a retrospective research design to describe the epidemic intensity, distribution, and trend of breast cancer in Shantou, Guangdong Province, Southern China from 2006 to 2017.

### 2.2 Data collection

The data, from the years 2006–2017, gathered from the Shantou's Medical Security Bureau covers the whole districts of Shantou including Jinping, Chenghai, Chaonan, Longhu, Haojiang, and Nanao districts.

### 2.3 Sampling

The study included a total of 34,121,556 insured patients. Of these, 5681 had breast cancer, including 5,655 women and 26 men. The inclusion criteria were as follows: (1) The International Classification of Disease (ICD) code of the cases was breast cancer, (2) The patients belonged to the local population, (3) The primary tumors were carcinoma of the breast, and (4) Information integrity was maintained. The exclusion criteria were as follows: (1) The patients did not belong to the local population, (2) Duplicate records. Further, the application information standard of the respondents was strictly defined and the regional population base index was obtained from the local official statistical yearbook.

The statistical calibers of breast cancer patients with medical insurance were as follows: (1) The types of medical insurance included staff, urban and rural residents, new rural cooperative medical system, and free medical service (a type of medical insurance for retired cadres and other special occupations), (2) People who died of breast cancer and were recorded in the medical insurance system in the mortality statistics, (3) The time period was from January 1, 2006 to December 31, 2017.

### 2.4 Data analysis

The data from survey items were recorded in Excel, and R software was used for statistical analysis. R version 4.0.1 (the R Development Core Team, Vienna, Austria) was used to develop the ARIMA and ETS models. A value of *p* < 0.05 was considered statistically significant. The ETS and ARIMA models were used to describe the regional distribution, time distribution, and population distribution of breast cancer in Shantou. The data were divided into 17 groups according to their age starting from 20 years old with an interval of 5 years, birth cohort (birth cohort equals period minus age) calculated to study the age, and the effect of period and birth cohort on female breast cancer.

### 2.5 Exponential smoothing (ETS) model

ETS technology is based on the method described by Hyndman *et al*.^19^ and implemented by the forecast package in the R software environment. We used the automatic selection of the ETS model to fit the exponential model of two parameters, and then we evaluated and selected the best performance model to simulate the data.^20^ The optimum model was chosen based on either the minimum Akaike information criterion (AIC), the corrected Akaike information criterion (AICc), or Bayesian information criterion (BIC).^21^ The Ljung–Box Q test was used to diagnose whether the residual error sequence was a white‐noise sequence.

### 2.6 Autoregressive integrated moving average (ARIMA) model

The Autoregression Integrated with Moving Averages (called the ARMA model) is the generalized model which has both Autoregressive (AR) and Moving‐average (MA) process elements. The ARIMA model is an extension of MA, AR, and ARMA models. We decided to build an ARIMA model to analyze the periodic data. The following steps are needed for the construction of this model. First, it needs to ensure that the time series is stable; if not, the non‐stationary time series can be made stable by difference. We use the Augmented Dickey–Fuller (ADF) test to realize the stationary hypothesis. If the result of the ADF test is significant, the sequence is stable. Second, the parameters of the ARIMA model are estimated by ACF and PACF. The ARIMA function was used to select the best ARIMA model according to either the minimum AIC, AICc, or BIC. Third, the goodness‐of‐fit of the model is evaluated, including statistical assumptions and prediction accuracy. If the model is suitable, the residuals are normal distribution and independent distribution (there should be no relationship between them). We use the Ljung–Box Q test to verify whether the estimated residuals meet the requirements of white noise sequences.

### 2.7 Performance statistic index

In order to evaluate the prediction performance of the models, the root mean squared error and the mean absolute percentage error were used to compare the prediction capabilities of the two models.

## 3 RESULTS

### 3.1 Demographic characteristics

**FIGURE 1:**
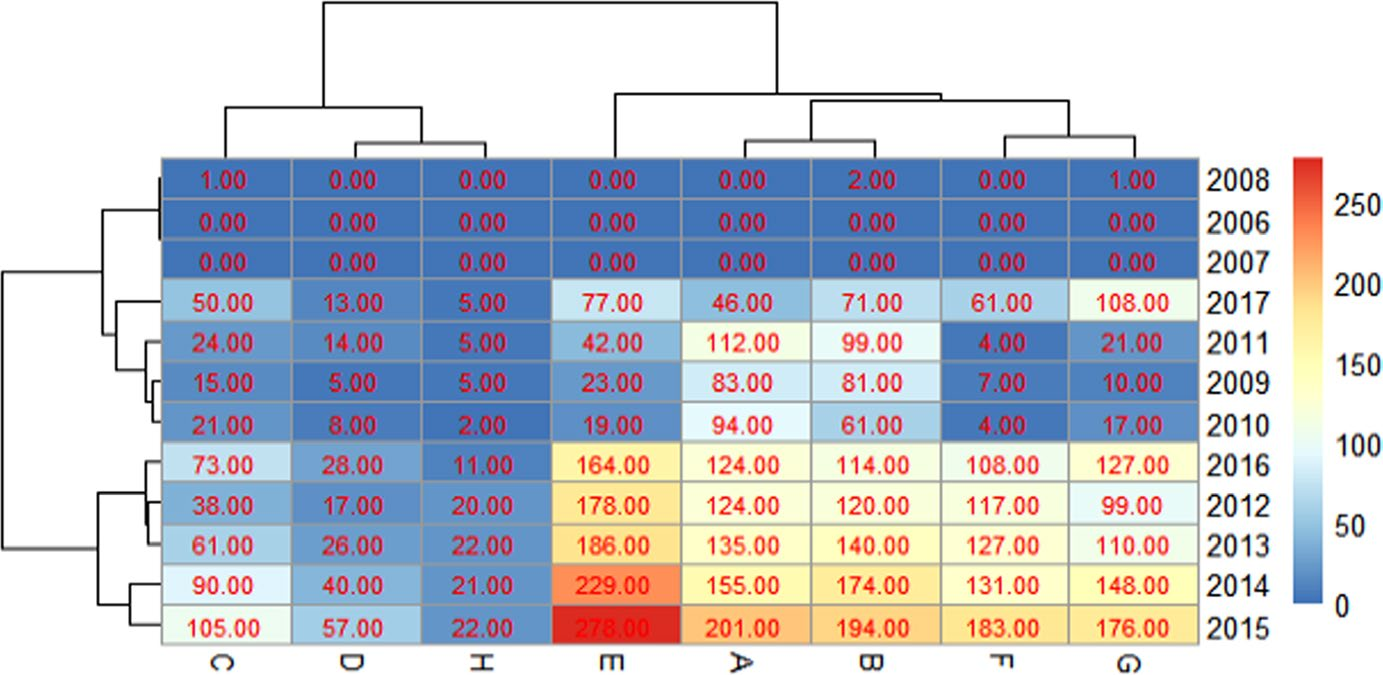
The new cases of Breast cancer in different districts of Shantou from 2006 to 2017.

Our study included 5681 patients who had breast cancer between 2006 and 2017. The incidence of breast cancer was highest in 2015 (Figure 1). The male‐to‐female ratio of the breast cancer patients was about 1:107 (the same ratio of the insured population was 1:1). Female patients accounted for 98.61% of the total insured population. The number of patients in the 50–59 age group was the largest. The incidence and the mortality of female breast cancer were 16.42/100,000 and 0.66/100,000, respectively (Table 1).

**TABLE 1:** Demographic characteristics of breast cancer patients from 2006 to 2017.

Variable	2006 n = 0	2007 n = 0	2008 n = 4	2009 n = 229	2010 n = 225	2011 n = 321	2012 n = 713	2013 n = 807	2014 n = 987	2015 n = 1216	2016 n = 749	2017 n = 430
Gender												
Male	0	0	0	1	0	1	5	5	7	5	2	0
Female	0	0	4	228	225	320	708	802	980	1211	747	430
Age												
0–9	0	0	0	0	0	0	0	0	0	0	0	0
10–19	0	0	0	0	0	0	1	1	0	0	0	0
20–29	0	0	0	1	0	0	12	10	10	13	6	1
30–39	0	0	2	22	18	30	57	82	103	106	61	46
40–49	0	0	2	66	65	101	231	246	285	240	229	135
50–59	0	0	0	94	91	120	241	296	276	436	261	139
≥60	0	0	0	45	51	70	171	172	313	421	192	109

As the chart shows, the trend for the incidence rate of female breast cancer was basically consistent with that of the total insured population, showing an M‐shape from 2006 to 2017. The first peak appeared in 2009 (59.07/100,000) and a second small peak appeared in 2015 (45.50/100,000), followed by a significant decrease (Figure S1).

### 3.2 Incidence of female breast cancer patients among different birth cohorts

The age‐specific incidence of female breast cancer was observed in different birth cohorts. The result showed that the incidence of breast cancer in each birth cohort increased significantly with age, reaching the peak at the age of 50–55, followed by a decreasing trend with further aging (Figure S2).

### 3.3 Result and test of the forecasting model

**FIGURE 2:**
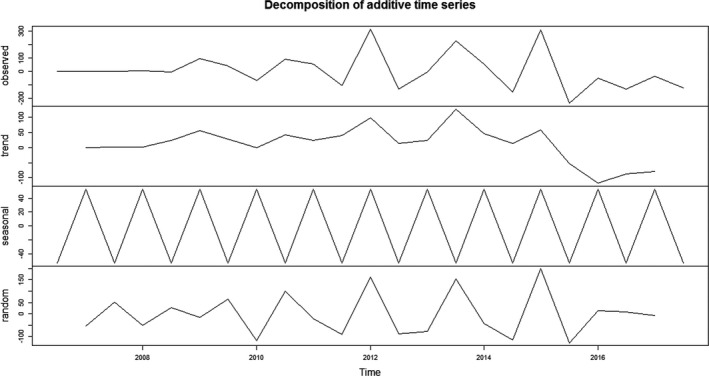
Incidence and changes in breast cancer every 6 months from 2006 to 2017.

Every six months from January 2006 to December 2017, obvious periodicity and seasonality had been found in the incidence cases and trend of breast cancer through spatial‐temporal analysis (Figure 2).

**FIGURE 3:**
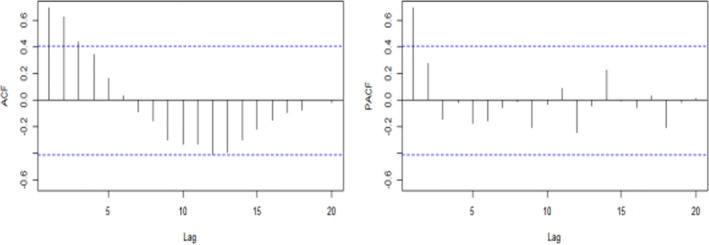
Time series autocorrelation and partial autocorrelation of breast cancer.

Ndiffs function showed that the time series of breast cancer do not need further differential (*d* = 0). ADF test results were statistically significant (*p* < 0.05), indicating that the series is stable. ADF and PACF were also used to estimate other parameters (Figure 3).

ARIMA (4, 0, 1) (0, 1, 0) was considered the best fit model when the ARIMA model was constructed using Time Series Modeler in the specific sample data. The Ljung–Box Q test showed that the residual sequence contains white noise (χ^2^ = 0.001, *DF*=1, *p* = 0.9737). The Ljung–Box Q test in the ETS model further showed that the residual sequence contains white noise (χ^2^ = 0.1147, *DF*=1, *p* = 0.7348). According to the ARIMA (4, 0, 1) (0, 1, 0) and ETS (M, N) models, the forecast results from June 2018 to December 2022 are as shown in Table 2.

**TABLE 2:** Forecast results of breast cancer from June 2018 to December 2022.

Date	ARIMA (4, 0, 1) (0, 1, 0) model forecast (95% CI)^+^	ETS model forecast (95% CI)^—^
June 2018	295 (113, 475)	200 (81, 482)
December 2018	136 (86, 358)	198 (142, 538)
June 2019	257 (67, 545)	196 (193, 586)
December 2019	196 (170, 561)	194 (89, 628)
June 2020	251 (172, 673)	192 (117, 666)
December 2020	174 (126, 633)	190 (143, 700)
June 2021	295 (230, 821)	188 (168, 733)
December 2021	159 (150, 712)	186 (181, 763)
June 2022	279 (109, 873)	184 (173, 792)
December 2022	191 (147, 602)	182 (178, 819)

+ ARIMA (4, 0, 1) (0, 1, 0) model: AIC=276.3, AICc=272.7, BIC=269.4; Coefficients: ar1 (0.1434), ar2 (−0.0836), ar3 (0.5796), ar4 (−0.3365), ma1(0.5726). ^—^ETS model: Smoothing parameters: alpha: 0.6749, beta:0.1203, gamma: FALSE; Coefficients: a = 202.2433, b = −2.0000.

**FIGURE 4:**
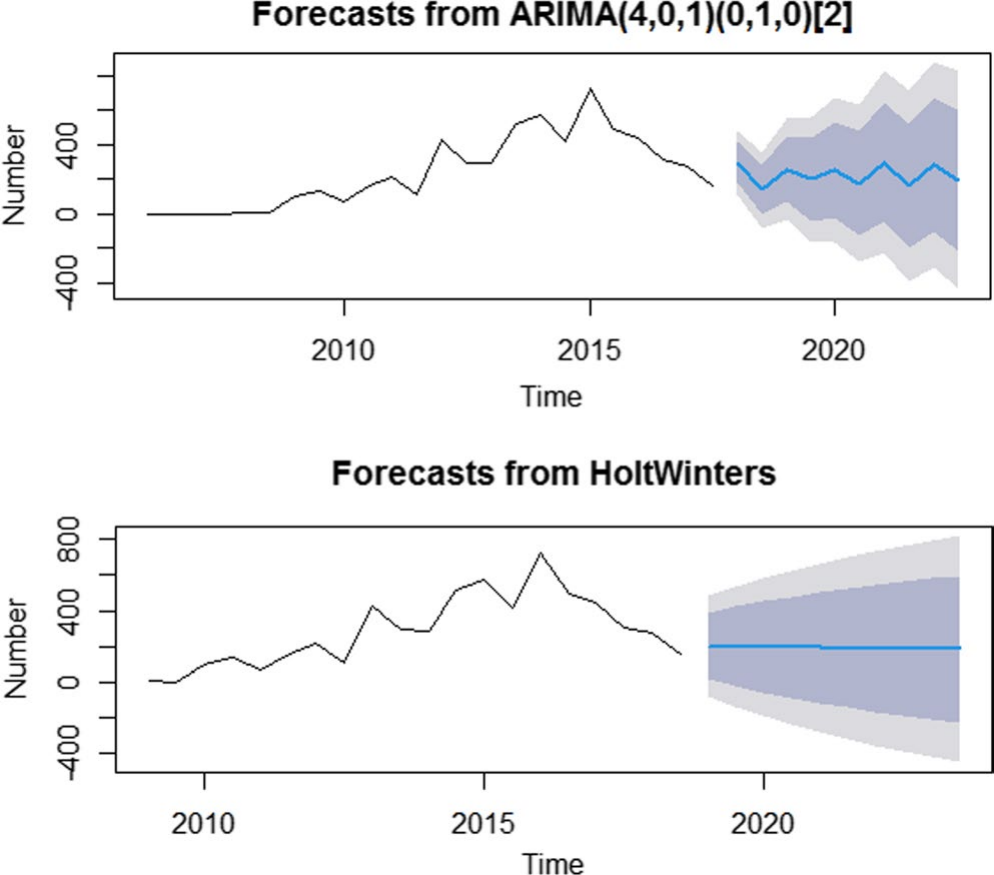
Trend chart of new incidence cases of breast cancer by ARIMA and ETS models.

Based on the ARIMA model and ETS model, the charts of breast cancer incidence showed a gradually decreasing trend expected in the future (Figure 4).

Comparing the performances of the ARIMA model and ETS model, ARIMA (4, 0, 1) (0, 1, 0) model had a lower the root mean squared error and the mean absolute percentage error than ETS (M, N) model.

## 4 DISCUSSION

This study showed that the incidence and the mortality of female breast cancer in Shantou were 16.42/100,000 and 0.66/100,000, respectively. Shantou's population development situation was similar to Guangdong Province. With the slow growth of the total population, fewer and better births have become the mainstream social fertility concept, and fertility willingness of the people has gradually declined. According to the *Population Development Plan of Shantou City (2018–2035)*, the birth rate was only 15.37‰, and the registered population aged 65 or more accounted for 8.89% of the total population by the end of 2017. Moreover, the average life expectancy was 76.3 years old, which is gradually increasing. The scientific and cultural quality and medical resources are constantly improving and people are paying more attention to tumor screening and treatment. The above factors may partially explain the much higher incidence rate than the mortality rate of breast cancer in Shantou.

The trend for the incidence of female breast cancer showed an “M” curve in the past decade. The reasons for the two peaks in 2009(59.07/100,000) and 2015(45.50/100,000) may be as follows: (a) *A notice from the State Council on printing and distributing the recent key implementation plan of the medical and health system reform (2009–2011)* required that all residents in urban and rural areas should be covered by employee medical insurance, resident medical insurance, and new rural cooperative medical system within three years, and the coverage rate should be increased to 90%. As a result, the rate of medical insurance and the detection rate of breast cancer increased. (b) Since 2015, the municipal organs, people's organizations, enterprises, and institutions in Shantou had organized a regular gynecological health examination (including cervical carcinoma and breast cancer examination) for female employees, which occurred once a year or once every 2 years. As a result, the detection rate of breast cancer among staff and workers increased significantly.

The result of the age‐specific incidence of female breast cancer indicated that 50–55 was a high‐risk age group. In China, several studies also showed that the trend of breast cancer incidence indicated peaks in the 45–55 years, 50–54 years, and 55–59 years age groups.^22^, ^23^ All forecasting models promoted that incidence of breast cancer is expected to have a gradually decreasing trend in the future.

### 4.1 Reasons for no special study on breast cancer in Shantou

Issues faced by epidemiological researches must be addressed, including narrow coverage, short time span, small sample size, and bad representativeness. Among all epidemiological studies of breast cancer, none have overcome all of these problems. Hence, obtaining all information regarding the whole population is impossible. Breast cancer is distributed among patients with different characteristics, which varies with the survival activities and time, such as lifestyle and medical treatment. Due to the limitation of manpower, funds, time, and cooperation in the implementation of the project, the investigation or interview of breast cancer patients without omission, gathering of all information, and implementation of random population sampling for breast cancer patients in the whole region is impossible. Implementation of the random population sampling can be time‐consuming, laborious, and prone to errors and omissions; thus, it is also an important reason why reports on the epidemiological research of breast cancer in Shantou are not performed.

### 4.2 Representativeness of the research

In order to fill the gap in academic knowledge of breast cancer epidemiology in Shantou, it is imperative to carry out such studies. Thus, to obtain the sample and to ensure the sample representativeness were key points of this study. Our study obtained ethical clearance and official permission from the Shantou's Medical Security Bureau. The data retrieved from the Medical Security Bureau in Shantou had high timeliness, authenticity, and integrity. China issued the notice of the State Council on the recent key implementation plan of medical and health system reform, requiring that the basic medical insurance for employees, residents, and the new rural cooperative medical system (NRCMS) should cover all urban and rural residents, as well as to attain an insurance coverage rate of more than 90%. According to Shantou Health Statistics Annual, the insurance coverage rate of the Shantou population reached 90% in the time period investigated in this study. Above all, the data obtained from the Shantou's Medical Security Bureau are representative.

### 4.3 The incidence rate of breast cancer in Shantou

This is the first analysis of the epidemiological characteristics and forecast of incidence using data from breast cancer patients in Shantou from 2006 to 2017. The data showed the epidemiology of breast cancer in the coastal areas of Guangdong Province, and the results could be compared with other areas in China and Asia. Our research could also help solve the main problems faced by epidemiological researchers of breast cancer in Shantou innovatively, met the needs of the project, and ensured the authenticity and reliability of the research conclusion.

The incidence rate of breast cancer in Shantou was 16.42/100,000 from 2006 to 2017. The latest report shows the cancer incidence of Guangdong Province ranks higher than the other provinces in China and breast cancer was the most common cancer in Guangdong Province. In 2013, the incidence rate of breast cancer in Shantou (32.18/100,000) was lower than that of Guangdong Province (40.99/100,000) but higher than that of China (28.42/100,000).^24^ According to the latest data, the cancer incidence in Shantou (45.50/100,000) was slightly higher than that in China (45.29/100,000) in 2015. The latest data also show that breast cancer is ranked as the fifth most common cancer in China. Compared with the national and Guangdong situation, the situation of breast cancer prevention and control in Shantou is still significant and should be addressed.^25^

### 4.4 Factors on the prediction results

The results of forecasting models showed that new incidence cases of breast cancer will slow decrease in the future, which is in contrast with the forecast results of McGuire's and Li Ni's studies.^5^, ^26^

The new guidelines of the *Healthy China initiative and promote people's health (2019–2030)* suggests that the measures should change from “curing disease” to “keep health,” from “treat the disease” to “preventive treatment of disease,” and strengthen the importance of early diagnosis and early treatment. China launched the “Cancer Screening Program” in 2012, which aimed to carry out the work of high‐risk population assessment, clinical screening, and early diagnosis and treatment of the five major types of cancer with high incidence including lung cancer, breast cancer, colorectal cancer, upper gastrointestinal cancer (esophageal cancer and gastric cancer), and liver cancer.^8^ This is an essential measure to reduce the incidence and mortality of breast cancer.^27^

Overall, Shantou may not be a high incidence area of breast cancer in China, and the cancer screening program promotes early diagnosis and early treatment in reducing the incidence and mortality of breast cancer.^28^ Moreover, the delayed treatment‐seeking behavior of patients in Shantou was observed. The factors that affect the delay of treatment seeking include hygienic knowledge from religious superstition, economic hardship, herbal tea intake, self‐medication in the absence of a medical professional, and healing prayer to Buddha,^29^ contributing to the gradually decreasing trend in new cases of breast cancer. Other factors that influence the incidence rate of breast cancer in Shantou are as follows:

(1) Diet factors: Local people have the habit of eating salted seafood, salted vegetables (such as pickles, dried radish), and fish sauce. These foods contain mildew, benzopyrene, and nitrosamines, which are carcinogens. Local people also have the habit of eating hot food and drinks, which may be a high‐risk factor for cancer.^30^, ^31^

(2) Smoking and drinking: Cigarette smoke contains a large number of carcinogens, with a clear carcinogenic effect. Reynolds et al. found that the incidence rate of breast cancer among smokers is higher than among non‐smokers.^32^ Some studies showed that women who drank alcohol seven or more times a week had an increased risk of breast cancer.^32^, ^33^

(3) Aging: In the past ten years, population aging has been accelerated due to changes in lifestyle, and it is an important factor in the rise of chronic diseases and the primary cause of high incidence of cancer.^32^, ^34^, ^35^

With the development of the social economy and the increase in urbanization, the fertility behavior, lifestyle, and diet of people have changed, including a decline in fertility rate, a reduction in breastfeeding, overweight and obesity, high energy and high‐fat diet, long‐term poor sleep quality, sleep deficiency, mental work pressure, excessive fatigue, emotional instability, and other factors, which increase the risk of breast cancer in women.^32^, ^34^, ^35^

### 4.5 Suggestion

The following interventions are suggested for the prevention of breast cancer:

(1) According to the risk factors and local conditions related to breast cancer, health education and promotion should be carried out through the collaborative participation of the government, community, and family.

(2) Self‐care and health management model should be advocated, with lifestyle intervention as the core, focusing on reasonable diet, appropriate exercise, and regular physical examination to enhance the awareness of the risk of breast cancer prevention in public.

(3) The medical treatment alliance, medical communities, and health services of the community family physician model should be combined and promoted, so that the public can access comprehensive, continuous, and effective basic medical and health services. These measures can improve the convenience of diagnosis and treatment, as well as the immediate and good healthcare‐seeking behavior of the public.

### 4.6 Limitations

Although we obtained new findings, there were several limitations of this study. Due to the limitation of manpower, funds, time, and cooperation in the implementation of the project, it was hard to collect the data of cancer cases from all hospitals in Shantou. Compared to the special cancer registration system, Medical Security Bureau system in Shantou lacks some relevant information on cancer patients. Moreover, there is the possibility of underreporting in cancer registration, which may affect the quality of the cancer registration system and the accuracy of the prediction model. Last, this study focused on analyzing the epidemiology of breast cancer alone. The comprehensive epidemiological analysis of other cancers is not available at this time.

## 5 CONCLUSIONS

This is the first population‐based retrospective study of breast cancer in Shantou, Guangdong Province of Southern China in the past ten years. The result of the age‐specific analysis of the incidence of female breast cancer indicated that the 50–55 years was a high‐risk age group. Based on the results of this study, it is recommended that the healthcare administration should strengthen programs for awareness and education regarding breast cancer prevention and control. It is also possible that feasibility of extrapolating the current methodology to other future studies or broader populations in which the cancer registry data are not available.

## 6 DECLARATION OF COMPETING INTEREST

The authors declare that they have no conflict of interest.

## 7 ETHICAL CONSIDERATIONS

Ethical clearance and official permission were obtained from the Shantou's Medical Security Bureau (Approval number: 2018087). Our research was conducted with the assurance that all of the information gathered would be treated with strict confidentiality and used solely for the study.

## AUTHORS’ CONTRIBUTIONS

All authors discussed the study design. Huang Lin, Lei Shi, Jiachi Zhang, and Jinchan Zhang analyzed the research data; Huang Lin, Lei Shi and Chichen Zhang wrote the first draft of the manuscript. Chichen Zhang edited the paper. Huang Lin and Lei Shi revised the manuscript.

## Supporting information

Fig S1‐S2

## Data Availability

The data that support the findings of this study are available from the corresponding author upon reasonable request.
