# Supplementary material for: Epidemiological characteristics and forecasting incidence for patients with breast cancer in Shantou, Southern China: 2006–2017
Source: Cancer Med. 2021 Mar 16;10(8):2904–13. doi: 10.1002/cam4.3843 (PMC8026945; doi:10.1002/cam4.3843)
Supplement: Supplementary file 1 — Fig S1‐S2 [file CAM4-10-2904-s001.docx]

**Supplementary materials**


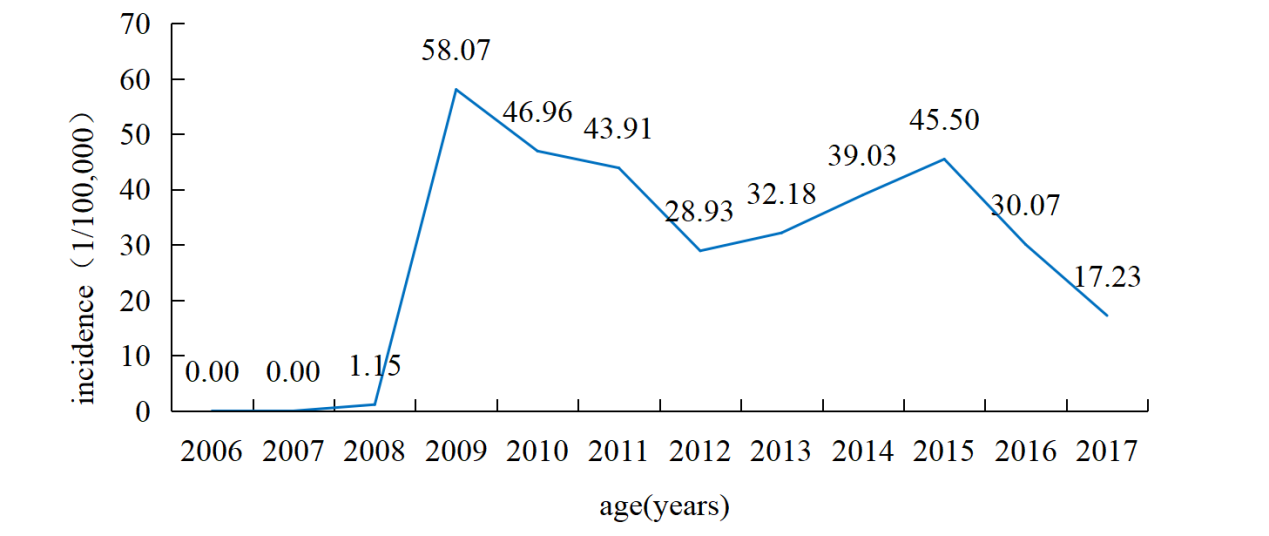


**Figure S1.** The incidence rate of female breast cancer in Shantou area from 2006 to 2017.

**
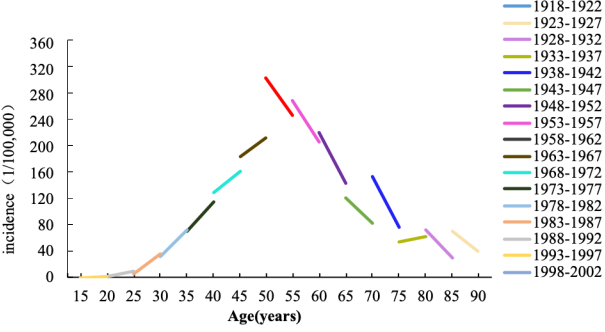
**

**Figure S2.** Age-specific incidence of female breast cancer in the cohort of 1922-1955.
